# Asbestos, Smoking and Lung Cancer: An Update

**DOI:** 10.3390/ijerph17010258

**Published:** 2019-12-30

**Authors:** Sonja Klebe, James Leigh, Douglas W. Henderson, Markku Nurminen

**Affiliations:** 1 Department of Anatomical Pathology, SA Pathology and Flinders University, Adelaide, SA 5042, Australia; 2 Asbestos Diseases Research Institute, University of Sydney, Concord, NSW 2139, Australia; jleigh@bigpond.com; 3 Department of Public Health, Faculty of Medicine, University of Helsinki, 00014 Helsinki, Finland; markstat.consultancy@elisanet.fi; 4 MarkStat Consultancy, 00250 Helsinki, Finland

**Keywords:** asbestosis, carcinoma, cumulative exposure, mesothelioma, multiplicative model, pathogenesis, smoking, synergism

## Abstract

This review updates the scientific literature concerning asbestos and lung cancer, emphasizing cumulative exposure and synergism between asbestos exposure and tobacco smoke, and proposes an evidence-based and equitable approach to compensation for asbestos-related lung cancer cases. This update is based on several earlier reviews written by the second and third authors on asbestos and lung cancer since 1995. We reevaluated the peer-reviewed epidemiologic studies. In addition, selected in vivo and in vitro animal studies and molecular and cellular studies in humans were included. We conclude that the mechanism of lung cancer causation induced by the interdependent coaction of asbestos fibers and tobacco smoke at a biological level is a multistage stochastic process with both agents acting conjointly at all times. The new knowledge gained through this review provides the evidence for synergism between asbestos exposure and tobacco smoke in lung cancer causation at a biological level. The evaluated statistical data conform best to a multiplicative model for the interaction effects of asbestos and smoking on the lung cancer risk, with no requirement for asbestosis. Any asbestos exposure, even in a heavy smoker, contributes to causation. Based on this information, we propose criteria for the attribution of lung cancer to asbestos in smokers and non-smokers.

## 1. Introduction

Asbestos exposure has been related to lung cancer causation since the 1930s, and much research has been published since then on epidemiological, clinical, biological, and medico-legal aspects of this relationship. Asbestos-related lung cancer is quantitatively more important than mesothelioma but is underrecognized because of the dominating effect of tobacco smoking in the causation of most lung cancers.

Four of the most important outstanding research questions in this area are: (i) How do tobacco smoking and asbestos fibers combine at a biological level to produce the well-known supra-additive interaction in causing lung cancer? (ii) How much asbestos exposure is needed to make a legally-significant contribution to lung cancer causation in the presence or absence of tobacco exposure? (iii) Is the presence of asbestosis necessary for any attribution of lung cancer to asbestos? (iv) How should lung cancer be compensated and how should tobacco smoking be allowed for in common law proceedings and in statutory compensation schemes?

This review considers the recent literature on causation and compensation of asbestos-related lung cancer and, in answering the above-mentioned medico-legal questions, proposes a rational, scientifically-based and equitable approach to compensation for asbestos-related lung cancer. The original Helsinki Criteria and updates did not consider smoking, and the exposure criteria were intended to apply irrespective of smoking, but here we have added a special consideration for life-long nonsmokers and ex-smokers with >30 yr since quitting.

### Preliminary Remarks

As reviewed by Henderson and Leigh [1], anecdotal autopsy reports of lung cancer in workers with asbestosis were reported in the mid-1930s [2,3,4,5], and in 1938, three German papers and a review from Austria reported evidence of a link between asbestosis and lung cancer [6]. Nordmann and Sorge referred to this occurrence as the occupational cancer of asbestos workers, and they thought that approximately 12% of asbestosis patients might develop lung cancer [7]. Nordmann and Sorge induced lung tumors in mice by the inhalation of chrysotile asbestos; the apparatus used for this experiment is illustrated in Proctor’s book The Nazi War on Cancer, and in 1943, the German government designated lung cancer in association with any degree of asbestosis as a compensable disease [6,8]. This nexus was rediscovered by Doll, who found that: One hundred and thirteen men who had worked for at least 20 years in places where they were liable to be exposed to asbestos were followed up, and the mortality among them compared with that which would have been expected on the basis of the mortality experience of the whole male population [9].

Thirty-nine deaths occurred in the group, whereas 15.4 were expected. The excess was due to excess deaths from lung cancer (11 against 0.8 expected) and respiratory and cardiovascular diseases with asbestosis. All the cases of lung cancer were confirmed histologically, and all were associated with the presence of asbestosis; the average risk among men employed for 20 or more years was of the order of 10 times that experienced by the general population. The risk became progressively less as the duration of employment under the old dusty conditions decreased.

In his paper, Doll, also placed emphasis on Merewether’s 1949 observation that at autopsy, lung cancer was found in 31 of 235 cases of asbestosis (13.2%), but in only 91 out of 6884 cases of silicosis (1.3%), and on Gloyne’s analogous observation of lung cancer in 17 of 121 autopsy cases of asbestosis (14.1%), in comparison to 55 out of 796 cases of silicosis (6.9%) [9,10].

The literature on asbestos and lung cancer has been reviewed extensively by Henderson, Leigh and coauthors [11,12,13,14,15,16,17,18,19,20,21]. From that literature, there is general agreement on a number of issues concerning asbestos and lung cancer, namely:

- All commercial asbestos fiber types can be implicated in the causation of lung cancer, e.g. amphibole anthophyllite and including the noncommercial amphibole, tremolite. In this context, commercial amphiboles crocidolite and amosite appear to be about equipotent on a fiber-for-fiber basis for lung cancer induction, and chrysotile is also implicated, especially in the chrysotile textile industry [22,23]. (There remains an unexplained 30-fold to 50-fold differential in the risk of lung cancer among the Charleston textile workers who used commercial Canadian chrysotile almost exclusively, in comparison to a much smaller risk for the Quebec chrysotile miners and millers).
- Whilst histological criteria have been established, the differential diagnosis of malignant mesothelioma, primary lung carcinomas, and pleural metastases can cause problems, especially in the case of sarcomatoid tumors [24,25]. A comparison of the frequency of DNA copy number changes between mesothelioma and lung carcinoma using discriminant analysis suggests that they are genetically-different tumors [26]. In the case of malignant mesothelioma, asbestos is overwhelmingly the singular identifiable causal factor, with only rare cases related to other factors such as erionite or fluoro-edenite fiber inhalation, ionizing radiation (sometimes in association with asbestos exposure), and innate susceptibility factors such as germline mutations affecting the BAP1 gene. Tobacco is not implicated in the causation of mesothelioma.
- Lung carcinoma, on the other hand, is a multifactorial cancer for which tobacco (especially cigarette smoke) represents the most potent causal factor on a worldwide basis. There are other known causes such as ionizing radiation (including radon gas daughters); certain metals such as hexavalent chromium, nickel, cadmium, arsenic and beryllium; silica, diesel particulate, and heated cooking [27].
- There are no clinical, radiographic or pathologic features of the tumor that discriminate clearly between lung cancers for which asbestos exposure can be implicated versus those for which it cannot; that is, there are no differences in the anatomical distribution of lung cancers in asbestos-exposed individuals (such as the upper vs. lower lobe or a central vs. peripheral localization), and all major histological types of lung carcinoma occur in asbestos-exposed individuals in comparison to nonexposed subjects, with no significant differences in the immunophenotypes, and no clear or diagnostic differences in the molecular-genetic profiles (see later discussion).
- The relationship between asbestos and lung cancer in general is governed by a near-linear dose-response relationship, with no clearly delineated threshold, but the gradient of the dose-response line is less steep than the analogous dose-response line between asbestos and pleural malignant mesothelioma. From Gustavsson’s meticulous case-referent studies in Stockholm County [28,29], there appears to be some evidence that the dose-response gradient for lung cancer is steeper at low cumulative exposures than at higher exposure [29] (see also [17,30], and later discussion in this review). In their 2000 review of 17 cohort studies, Hodgson and Darnton commented that if a threshold does apply to lung cancer induction by amphibole asbestos, ‘it must be very low’, where as a threshold for chrysotile—a ‘zero or at least very low risk’—is ‘strongly arguable’ (but they commented that the asbestos-related dose-response effect for lung cancer in the Charleston textile cohort is ‘untypically high’ [31]). In contrast, some other studies have also found a higher dose-response effect for asbestos textile workers than for other chrysotile exposures [32,33].

However, several longstanding issues on which dissenting conclusions have been drawn are the exact nature of interaction between tobacco smoke and asbestos, and whether asbestosis is a requirement for the attribution of lung cancer to asbestos in an asbestos-exposed smoker:

- It is accepted that tobacco (especially cigarette) smoke and asbestos functionally interact in the causation of lung cancer; however, the type and strength of this interaction have occasionally been the issue if debate [34]. It is our opinion that the effect is synergistic, i.e., the combined effect is greater than the sum of the individual effects. By definition and in biological terms, the difference (the synergistic effect) cannot be apportioned back to each of these individual carcinogens; this issue is explored further in a later section of this review.
- It is our opinion on the balance of probabilities that asbestosis is not a necessary prerequisite for the attribution of lung cancer to asbestos in an asbestos-exposed smoker.

The following section of this review highlights a few publications on this issue.

## 2. Materials and Methods

The authors reviewed key epidemiological, pathological, and basic biology papers that had been published since the previous review by two of the authors on the subject [17]. A formal meta-analysis was not performed.

## 3. Discussion

### 3.1. Lung Cancer and Cumulative Asbestos Exposure, with or without Asbestosis-Source Epidemiological Data

A 2005 review limited to nine key epidemiological papers found that seven of the nine papers supported the case that asbestosis was not a necessary prerequisite for attribution of lung cancer to asbestos exposure; it found weaknesses in all the studies reviewed, and concluded that a consideration of fiber type would be critical in putting the issue beyond doubt, and that epidemiology alone could not resolve this medico-legal issue [35]. This review was limited to all the studies above and many others are analyzed in greater detail in the reviews mentioned above [17,30,35,36].

A study bearing on lung cancer mortality among insulation workers was reported by Markowitz and colleagues [37]; this investigation focused on 2377 male North American insulators for whom chest x-ray, spirometric, occupational, and smoking data were collected in 1981–1983, and for 54,243 nonasbestos-exposed blue collar male workers for whom occupational and smoking data were collected in 1982. Lung cancer caused 339 (19%) insulator deaths. The authors found that lung cancer mortality was increased by asbestos exposure alone among nonsmokers (rate (risk) ratio [RR] = 3.6; 95% confidence interval [CI] = 1.7–7.6) i.e., among those without evidence of asbestosis on chest x-ray at the beginning of the study, although 1.3% of this group died from asbestosis during the period 1981–2008), in comparison to asbestosis among nonsmokers (RR = 7.40; 95% CI = 4.0–3.7), and by smoking without asbestos exposure (RR = 10.3; 95% CI = 8.8–2.2). In this study, the joint effect of smoking and asbestos alone was additive (RR = 14.4; 95% CI = 10.7–19.4), and for asbestosis, the joint effect with smoking was supra-additive (RR = 36.8; 95% CI = 30.1–45.0). Insulator lung cancer mortality halved within 10 years of smoking cessation, and it approached that of never-smokers at 30 years after cessation. Unsurprisingly, the Markowitz et al. study has attracted criticism [38,39], to which Markowitz et al. responded [40]. In an editorial on the Markowitz et al. paper, Balmes commented that we ‘know’ that (i) ‘asbestos exposure alone is capable of causing lung cancer’; (ii) asbestos and smoking together are associated with at least an additive increased risk of lung cancer; (iii) the presence of asbestosis further increases the risk in both smokers and nonsmokers; and (iv) smoking cessation substantially decreases the lung cancer risk associated with asbestos exposure [italicized text in the original editorial]) [41].

A comprehensive study of lung cancer mortality in UK asbestos workers based on 1878 deaths in the period 1971–2005 confirmed a multiplicative interaction of tobacco and asbestos exposure, an increased dose-related risk of lung cancer in never-smoking asbestos workers compared to the never-smoking general population, based on 32 never smoker male lung cancer deaths (SMR 95% CI 93–192) (i.e., P ~ 0.06 one- sided), and a substantial reduction in lung cancer risk with smoking cessation [42].

A very large pooled analysis of 14 case—referent studies in Europe and Canada conducted in 1985–2010 (17,705 cases, 21,813 referents, 6958 exposed males, 482 exposed females)—showed no departure from a multiplicative model in males and a more than additive effect in females [43]. In all males, the mean coefficient was 0.061 per fiber-year, and for blue collar worker males, 0.033 per fiber-year. Females showed no significant increase in risk (*p* > 0.05), but exposures were much lower [43].

We also assign significance to the 1994 case-referent study reported by Karjalainen and colleagues on the association the between lung asbestos fiber burden and the risk of lung cancer—based on 113 lung cancer patients treated by surgery, vs. 297 autopsy referents in the Finnish population. Lung tissue fiber assays were carried out for fibers longer than 1 μm by SEM (mainly amphibole fibers). The odds ratio for lung cancer (OR_LCA_) increased to 1.7 for concentrations in the range 1.0–5.0 million fibers/g dry lung and to 5.3 for concentrations of ≥5.0 million fibers/g dry lung, in comparison to a reference group with a fiber concentration of <1.0 million [44]. The authors stated that when two cases of asbestosis and seven cases of minor ‘histological fibrosis compatible with asbestosis’ were excluded, an elevated OR_LCA_ was still associated with asbestos fiber concentrations of ≥5.0 million fibers/g dry lung (the age-adjusted OR_LCA_ was 2.8; 95% confidence interval, CI = 0.9–8.7; *p*-value = 0.07) and for fiber counts in the range 1.0‒5.0 million, the OR_LCA_ was 1.5 (95% CI = 0.8–2.9; *p* = 0.19). This study has been criticized because the results failed to achieve ‘significance’ in terms of *p* values, thereby proving that ‘significance’ lay only with the cases of fibrosis [45]. However, this criticism is weakened by two factors: (i) the limit *p* ≤ 0.05 is an arbitrary statistical convention, and reality often lacks sharp boundaries of this type; and (ii) what is important in this study is the trend from a low to a higher OR_LCA_ with transition from an intermediate fiber count (1.0–5.0); the clinical asbestosis cases were in the heaviest exposure group and that the mild histological fibrosis cases were in the intermediate exposure group—the OR_LCA_s then become 2.85 and 1.8 respectively, as consistent as possible with the age-adjusted OR_LCA_s of 2.8 and 1.5 in the original paper and trend testing then yields Χ^2^ (1 d.f.) = 7.2 (*p* < 0.01). In addition, one can recalculate the OR_LCA_ for adenocarcinoma only, after the exclusion of all cases with any fibrosis; assuming that all were in the high fiber group, the OR_LCA_ (2.65; 95% CI = 1.11–6.26; *p* < 0.001) is still significantly elevated, for a count >1.0 million compared to <1.0 million. The study by Karjalainen and colleges [44] study formed the basis in part for the uncoated amphibole asbestos fiber counts specified originally in The Helsinki Criteria [46].

An earlier review or meta-analysis of epidemiologic studies concluded that persons without asbestos-related pleural plaques do not have an increased risk of lung cancer in the absence of parenchymal asbestosis [47]. The reviewer inferred that this conclusion provided indirect supportive evidence for the proposition that asbestosis is a necessary precursor of asbestos-related lung cancer. Nurminen and Tossavainen showed that such a proposition lacked any logic [48,49].

In this context, it is our assessment that the weight of scientific evidence indicates that the risk (and occurrence) of lung cancer is related to cumulative asbestos exposure per se, with no requirement for asbestosis [1,17,28,29,31]. We draw this conclusion, although dissenting opinion this issue has persisted [50,51,52] for years after the Hughes and Weill study [53]. That study favored a requirement for asbestosis in order to attribute lung cancer to asbestos. But if asbestosis is present, the risk of lung cancer is further enhanced (for additional references on this issue and our responses to the ‘dissenting’ opinion, see Henderson and Leigh [1,17,30]. In this setting, we consider that asbestosis appears primarily to be a marker for substantial to heavy cumulative asbestos exposure.

### 3.2. A Peer Opinion Regarding the Requirement for Asbestosis for Attribution of Lung Cancer

The cumulative exposure model with no requirement for asbestosis was further strengthened by the results of an international ‘Delphi’ study on asbestos-related disorders, published in 2009 [54]. The database PUBMED was searched for all persons in the world with three or more first-author publications on asbestos-related disease for the period 1991–2002: 95 were found. The respondents to computer questionnaire (34/95) replied to a series of questions, one of which was ‘Pulmonary fibrosis is a prerequisite to attribute the development of lung cancer to asbestos’; there was strong consensus that this was not the case (median score 1 out of 10 on a scale of strongly disagree (0) to strongly agree (10). A second question was ‘Workers with asbestos exposure and pleural plaques or diffuse pleural thickening without (fibrosis) are at increased risk of lung cancer’; there was strong consensus that this was the case, with a median score 9 on the same scale. A third question was ‘Workers who have significant asbestos exposure (but who do not have asbestosis) are at increased risk of bronchogenic carcinoma’; there was strong consensus that this was the case (median score of 9 on the same scale). A fourth question was ‘A history of asbestos exposure of sufficient duration, dose and latency is likely to be the cause of interstitial fibrosis in the absence of other explanations’; a consensus was reached with a median score 9 on the same scale.

### 3.3. The Pathogenesis of, and Some Molecular Alterations in, Asbestos-Related Lung Cancer

The cellular and molecular pathogenesis of fiber-induced lung cancer has been extensively studied over the last 20–30 years, and although knowledge is incomplete, much is known about it. The current consensus view is that asbestos participates in both the initiation and the proliferation phases of tumor development. The monograph on human carcinogenesis by arsenic, metals, fibers, and dust [55] reviews the evidence and the Consensus Report states as much.

Carcinogenesis by fibers appears to be a multistage process which may arise by the ability of fibers to cause (i) altered expression or function of key genes arising from genetic or epigenetic alterations; (ii) altered cell proliferation; (iii) altered regulation of apoptosis; or (iv) chronic, persistent inflammation [55].

Our opinion, based on current scientific literature, is that in view of the capacity of asbestos fibers to be involved at all stages of tumor development, all cumulative exposure to asbestos in an individual plays some contributory part in causation of the tumor, and consequently, that this cannot be separated from the concurrent effects of tobacco smoke; the evidence suggests that asbestos fibers increase the uptake and metabolism of polycyclic aromatic hydrocarbons (which are amongst the best characterized carcinogens in cigarette smoke) by lung epithelial cells [56,57,58,59]. In addition, cigarette smoke increases the binding of asbestos fibers to lung epithelial cells [60,61]. The lung epithelial cells are genetically damaged, some damaged cells become malignant, and malignant cells proliferate at different times. DNA repair processes are occurring (or may be impaired), and oncogenes and suppressor genes are activated and inactivated.

**Figure 1:**
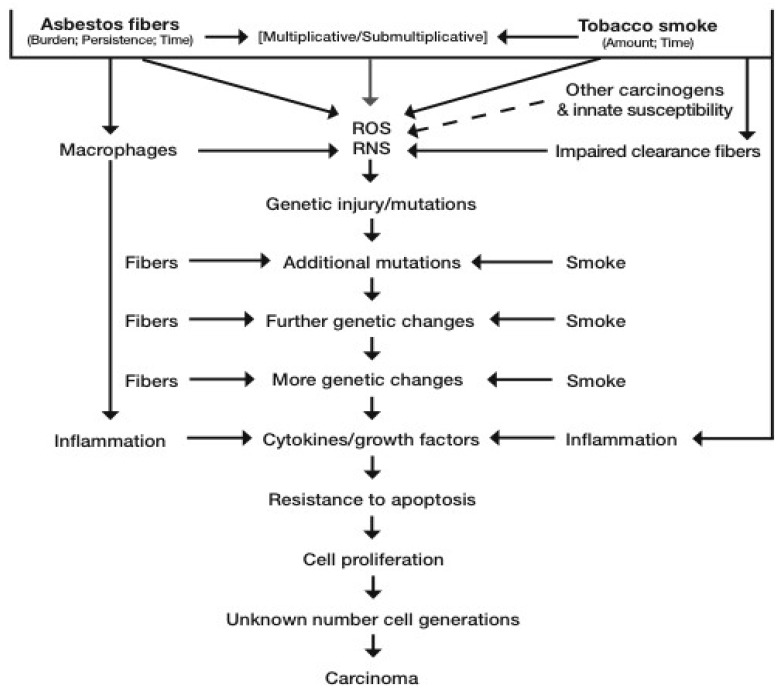
Mechanism of lung cancer causation by asbestos and tobacco smoke. ROS = reactive oxygen species; RNS = reactive nitrogen species.

Altered cells are being removed by apoptosis, necrosis, and immunological means. Fibers are being cleared at differing rates and, if exposure continues, they continue to be deposited in the lung. Components of cigarette smoke have been shown to impair the clearance of particulates, including asbestos fibers, form the upper airways [62,63]. All these processes at a cellular level are stochastic, in that probabilities of fiber-cell interaction depend on the number of fibers and the numbers of cells present at any point in time and space. Hence, simplistically, the more fibers, the more there are free radicals, and the greater the probability of genetically-damaged and proliferating cells at any given time point. This is shown diagrammatically in Figure 1. See also the Interational Agencyf (or Research on Cancer (IARC) Monograph 100C [55] for a detailed review of lung cancer causation by asbestos. Recently, a putative new mechanism has been proposed whereby asbestosis, lung cancer, and mesothelioma pathogenesis has a common ground based on asbestos-induced epithelial to mesenchymal transition (EMT), mediated through a transforming growth factor (TGF) β pathway. New direct evidence for this mechanism in bronchial epithelial cells exposed in vitro to chrysotile has recently been published [64].

To the best of our knowledge, the molecular profiles of the carcinomas (such as epidermal growth factor or anaplastic lymphoma kinase mutation expression) do not distinguish definitively between those lung carcinomas for which asbestos causation can be implicated, in comparison to those for which it cannot. Although a few recent studies have found an association between the type of mutation and asbestos causation, in our opinion, it is still not possible from genetic studies to clearly identify an asbestos-caused carcinoma in comparison to equivalent lung carcinomas in the general population. Nelson et al. found k-ras mutations more frequently in asbestos-exposed lung adenocarcinomas than carcinomas in nonexposed lung [65]; Kettunen et al. found a statistically significant greater frequency of chromosome position 2p16 loss in lung cancers (all cell types) from asbestos exposure compared to nonexposure carcinomas [66]. The 2014 update of the Helsinki criteria recommends no changes to the 1997 criteria in relation to lung cancer. The report stated that the only means to evaluate the potential usefulness of a molecular assay is by comparison with the present criteria of attribution, preferably in prospective international multicenter studies. It also noted that further studies are required before genetic biomarkers can be applied to support attribution in individual cases [67].

### 3.4. The Synergy between Asbestos Fibers and Tobacco Smoke for Lung Cancer Causation Epidemiological Data

A current consensus view is that asbestos exposure and tobacco smoking interact synergistically for the causation of lung cancer, described by a *multiplicative* model, recognized since 1968 [68], although a more than *additive* (submultiplicative) effect has also been invoked [69]. Furthermore, an additive model has been applied for the Quebec chrysotile cohort in particular, and Markowitz and colleagues recorded an additive and supra-additive effect for asbestos alone and asbestosis respectively in their mortality analysis of North American insulators [37] (see Appendix A for definition of the models).

In the very large 1979 cohort mortality study of US insulation workers (276 lung cancer deaths) by Hammond et al. [70], smoking increased the risk of lung cancer by about 10-fold, and asbestos increased the risk by about 5-fold; the two together increased the risk by about 50-fold (not 15-fold), compared with that of the nonsmokers who were unexposed to asbestos; an almost pure multiplicative effect. If all factors contributing to lung cancer were summarized in a figure of 53 to quantify the actual average risk in a smoker with asbestos exposure, its components are 1 for the base risk, 10 (10.85–1) for the smoking risk, 4 (5.17–1) for the asbestos risk, and 38 (53–1–10–4) for the risk that depends on the interaction between smoking and asbestos. It is readily evident that the interaction is of major importance.

However, from a pathobiological perspective and as matter of definition, the interactive effect cannot be partitioned into the individual effects of smoking and asbestos. Nurminen and Karjalainen have also emphasized the multiplicative proportion of fatalities related to occupational factors (including lung cancer with smoking and asbestos) in Finland [71]. The Australasian Faculty of Occupational Medicine of the Royal Australasian College of Physicians has reiterated the consensus view that the relationship is generally multiplicative, although recognizing that lighter smokers may have a greater dose-related risk of lung cancer from asbestos [72].

An authoritative review on possible biologic mechanisms for this synergistic effect and the forms of statistical interaction has been made by the International Agency for Research on Cancer [73]. After reviewing various studies, the authors concluded that the multiplicative model generally gives the best fit. Liddell argues that the Quebec miner and miller cohort studies indicate an additive effect of smoking and asbestos rather than a multiplicative one, although he concedes than an ‘additive hypothesis is not generally applicable’ [74,75]; he mentions the review of Lee [76] on the nature of the interaction between smoking and asbestos in causing lung cancer, but does not deal with Lee’s finding that in 30/31 datasets analyzed, the effect was greater than additive, with no overall departure from the multiplicative model. Lee analyzed the Quebec cohort data by two different methods, and found that the data did not show true departure from the multiplicative model; he concluded that the multiplicative model is the best overall fit. A large case-referent study (1004 cases and 1004 matched controls) found no statistical evidence to support departure from a multiplicative model [77]. Vainio and Boffetta concluded that the interaction approximates the multiplicative model, and that tobacco smoke and asbestos may have independent effects on the multistage process of carcinogenesis [73]. A recent meta-analysis including some more recent studies showed a supra additive synergy implying biological level interaction [69]. This is also supported by the recent large study by Olsson et al., where 14 case-control studies conducted in 1985–2010 in Europe and Canada were pooled. These included more than 17,000 lung cancer cases and 21,813 controls with detailed information on tobacco habits and lifetime occupations. An increasing lung cancer (all major histological types) risk for men was seen with increasing asbestos exposure in all smoking categories, whereas in women, lung cancer risk was increased in current smokers (ORs ~ two-fold), regardless of histological type. The data again supported a multiplicative effect of effect of combined asbestos exposure and smoking in males, and the effect was more than additive in females [43].

#### 3.4.1. The Synergy between Asbestos Fibers and Tobacco Smoke for Lung Cancer Causation: Biological Data

Tobacco smoke can act at early stages, inducing genetic alterations, DNA adducts, and mutations in genes critical to cancer formation. Tobacco smoke can also influence the uptake of particulates by bronchial epithelial cells [78]. It is likely that the chemical basis of tobacco-induced mutations is the oxidation of DNA groups by free radicals. Epidemiological evidence suggests that tobacco may also act at later stages of the cancer process. Tobacco smoke has both gaseous and particulate components; the latter can stimulate macrophages to release cytokines and free radicals [79]. Macrophages associated with tumors are critical to both the initiation and maintenance of lung cancers, as well as to metastases [80]. Asbestos can be cytotoxic (killing cells) and genotoxic (damaging genes), and may cause proliferative lesions in the lungs. Asbestos fibers can generate free radicals either directly or after attempted phagocytosis by macrophages. It is likely that the chemical basis of asbestos-induced mutations is the oxidation of DNA groups by free radicals. Asbestos is genotoxic to bronchial epithelial cells [45,65,81,82,83].

Asbestos may cause chronic inflammatory changes, which release cytokines (including growth factors), providing a selective advantage for cells which have undergone cancerous mutation due to carcinogens in the tobacco smoke or due to asbestos itself [73]. These cytokines and chemokines produced during the inflammatory process are critical to tumor development [84]. Recent studies have directly shown interaction between tobacco smoke and known lung carcinogens of the type found in tobacco smoke and asbestos in causing lung cancer [2,85,86,87,88]. The exact mechanisms that underpin the synergism between tobacco smoke and asbestos for lung cancer induction are not fully understood. It should be emphasized that the interaction happens at a biological level, and is not just a statistical interaction [89].

Several mechanisms have been proposed as potential explanations, of which the most likely appear to be the following:

(1) asbestos contributes to improved uptake of chemical carcinogens in cigarette smoke and their metabolism to carcinogenic metabolites in lung epithelial cells
(2) inhibition of clearance and retention of carcinogens
(3) chronic inflammation that drives development and metastases of lung tumors
(4) Direct synergistic effects on proliferation

For example, carcinogens in cigarette smoke such as benzo[a]pyrene (B[a]P) may be adsorbed onto asbestos fibers (e.g., crocidolite or chrysotile), with subsequent delivery of the carcinogens into cells at high concentrations [56]. Tobacco smoke may interfere with the clearance of asbestos from the lungs; Churg and Stevens found elevated concentrations of asbestos fibers in the airway tissues of smokers in comparison to nonsmokers, for both amosite (~6-fold) and chrysotile (~50-fold), especially for short fibers (in comparison, parenchymal amosite fiber concentrations were comparable in the smoker and nonsmoker groups) [90]. The importance of the inflammatory responses is reviewed in [84]. Direct supra-additive effects on mutations in bronchial epithelial cells of free radicals are generated by tobacco constituents and asbestos fibers. In this context, some carcinogens in tobacco smoke have the capacity to induce showers of free radicals (such as hydroxyl radical spin adducts [91]. This is the same effect induced by ionizing radiation [92] and asbestos fibers [20,93].

#### 3.4.2. The Synergy between Asbestos Fibers and Tobacco Smoke for Lung Cancer Causation–Animal Studies

Kimizuka et al. investigated the cocarcinogenic effects of chrysotile and amosite asbestos plus the tobacco smoke carcinogen benzo[a] pyrene (B[a]P) in hamster lungs [94]. Between 18–24 months after the last intratracheal instillation, the number of tumors was examined. In the chrysotile + B[a]P group, there were 37 tumors that included 16 carcinomas in 12 animals, and 30 tumors including 11 carcinomas in 12 animals were found in the amosite + B[a]P group. In the chrysotile + B[a]P group, all animals developed tumors, as did 92% in the amosite + B[a]P group; the carcinomas were found in 83% and 67%, respectively. The numbers of tumors and carcinomas and the frequency of the tumor-bearing or carcinoma-bearing hamsters in the chrysotile + B[a]P and the amosite + B[a]P groups were significantly higher than those of the groups instilled independently. Although the number of tumors or the frequency of tumor-bearing animals was higher in the chrysotile + B[a]P than the amosite + B[a]P group, the differences were not significant. The results were considered to indicate that both chrysotile and amosite play an important role in the genesis of bronchogenic carcinoma.

Harrison et al. investigated the combined effect in the lungs of rats of simultaneous exposure to chrysotile asbestos and N-nitrosoheptamethyleneimine (NHMI) for the development of metaplastic, hyperplastic, and neoplastic lesions [85]. The effects were more pronounced in males than females, and NHMI administration increased the frequency of hyperplastic lesions with augmentation by chrysotile (although this was not statistically significant), but a ‘promoting’ effect of chrysotile was observed for the induction of lung tumors (all but two out of 11 primary tumors were in rats treated with both NHMI and chrysotile), although this finding was not confirmable statistically because of the small number of tumors observed. A sex difference in inflammatory responses involving higher expression of Cox-2, VEGF-A, and VEGF-R2 in males compared with females has recently been observed in other models [95], and consequently, research bodies now recommend use of sex-matched cohorts [96].

Kamp et al. [82] found evidence that cigarette smoke extracts (CSE) augmented amosite asbestos-induced alveolar epithelial cell (AEC) injury by generating iron-induced free radicals that damage DNA. In this study, amosite or CSE each resulted in dose-dependent toxicity to AECs (WI-26 and rat alveolar type I-like cells), and the effects of CSE + amosite in combination were synergistic in A549 cells and additive in WI-26 cells. The authors concluded that the data adduced further support for genotoxicity of asbestos and cigarette smoke in relevant target cells in the lung, and that iron-induced free radicals may, in part, cause these effects.

Loli et al. investigated the mutagenic effects of amosite asbestos and B[a]P in the lungs of λ-lacI rats. In the first experiment, intratracheal instillation of both amosite and B[a]P in combination resulted in a supra-additive increase in mutation frequency, in comparison to rats treated only with amosite or with B[a]P [86]. In the second experiment, the intraperitoneal administration of B[a]P did not alter significantly the mutation frequency induced by amosite after either four or 16 weeks of treatment, and B[a]P-DNA adduct levels were unaffected by amosite cotreatment in both experiments. The authors concluded that the ‘striking enhancement effect of B[a]P may provide a basis for understanding the suspected [sic] synergism of smoking on asbestos carcinogenesis.’ This group also reported in 2006 that DNA adducts induced by simultaneous B[a]P and man-made mineral fibers (MMMF) indicated a strong increase in the mutation frequency [97].

A very recent study in a mouse inhalation model shows that exposure to combined tobacco smoke and chrysotile asbestos suppressed the innate immune response (NLRP3 inflammasome) to asbestos fibers, resulting in reduced fiber clearance and more chronic inflammation, leading to carcinogenesis. Confirmatory in vitro studies in human monocytes produced a similar effect with combined tobacco smoke and asbestos exposure [98].

#### 3.4.3. The Synergy between Asbestos Fibers and Tobacco Smoke for Lung Cancer Causation—Studies in Humans

In addition, a recent genome wide methylation study (using Illumina HumanMethylation450K Bead Chip) of lung cancer tissue and paired normal lung from 28 asbestos-exposed or nonexposed patients revealed that differential methylation profiles could be identified, depending on the etiology; the authors suggested that the methylation changes in tumors may be specific for risk factors [99]. At this stage, this is early and preliminary work, but it is conceivable that in future, mutation signatures associated with different etiologies of lung tumors may be able to verify the mutagenic effect of smoking and/or asbestos exposure in the in causal pathways in the tumor tissue.

**Figure 2:**
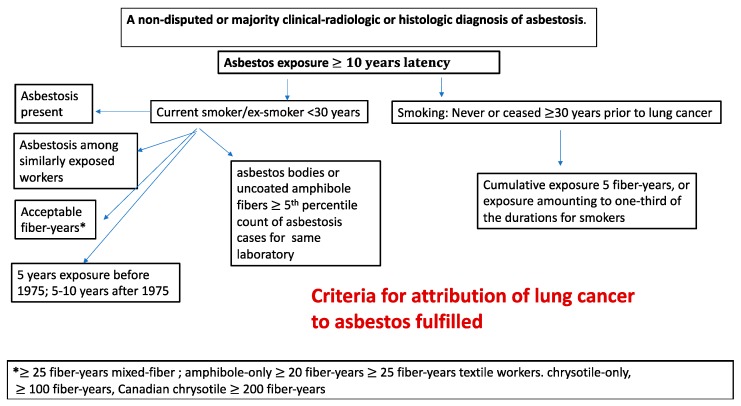
Simplified flow chart for attribution of lung cancer to exposure to asbestos, taking into account tobacco smoke.

In addition, molecular studies in humans ‘further suggest that asbestos enhances the mutagenicity of tobacco carcinogens and that it acts, at least in part, independent of the tissue damage responsible for fibrosis.’ [87] Nelson et al. [65] investigated k-ras codon 12 mutations among 84 male patients with adenocarcinoma of lung for whom a work history was available, as well as a chest radiograph for all those who had a history of occupational exposure to asbestos. K-ras mutations were more prevalent in patients with a history of occupational asbestos exposure (crude OR = 4.8; 95% CI = 1.5 − 15.4) in comparison to those without asbestos exposure. The association remained after adjustment for age and pack-years smoked (adjusted OR = 6.9; 95% CI = 1.7 − 28.6). An index score that weighted for both the dates of exposure and estimated exposure intensity indicated that subjects with k-ras mutations had significantly greater asbestos exposures than those without such mutations (*p* < 0.01). A supra-additive effect of smoking and asbestos exposure on the percentage of k-ras mutations was shown (Figure 2 in the original). The duration of exposure was not associated with k-ras mutations, but time after initial exposure was significantly associated with mutation status. ‘The association between k-ras mutation and reported asbestos exposure was not dependent on the presence of radiographic evidence of asbestos-related disease.’ The findings were considered to suggest ‘that asbestos exposure increases the likelihood of mutation at k-ras codon 12 and that this process occurs independently of the induction of interstitial fibrosis.’ In an earlier study, the same group of researchers also found that asbestos exposure (*p* < 0.01) and a duration of more than 50 years of smoking (*p* < 0.01) were significantly associated with exon deletion from the fragile histidine triad (FHIT) gene [100].

#### 3.4.4. The Synergy between Asbestos Fibers and Tobacco Smoke for Lung Cancer Causation: Summary 

While the precise mechanism of interaction between asbestos exposure and tobacco smoke in causing lung cancer is not fully understood, a great deal of research has been done in this area over the last 20–30 years. It is not possible in our view to analyze their causal interdependence in the individual case when both have acted, as they must do to some extent, from a purely physico-chemical point of view, and it is thus more probable than not that in this situation, the lung cancer was the singular result of the two factors acting together.

Exposure to either factor alone without the presence of the other is capable of causing lung cancer, but when both are present, in our opinion, on the basis of the above biological evidence of interdependence, both must have been acting to some extent in the process of carcinogenesis. To assume otherwise would be to reject the existence of physical and chemical laws governing biological behavior. All medical science depends on the transfer of knowledge gained from cellular, molecular, and animal experiments to man, without requiring the replication of the chemistry and physics in the human by direct invasive human experimentation. A corollary of the above is that any asbestos exposure, even in a heavy smoker, could be considered to legitimate causation.

Our current understanding of the biological mechanism of lung carcinogenesis induced by asbestos and tobacco is shown in Figure 1.

### 3.5. Relevance of Estimates of Cumulative Asbestos Exposure to Causal Attribution and Lung Cancer Risk

A cumulative exposure of 25 fibers/mL-years (fiber-yrs) can be associated with the first signs of clinical asbestosis [9], although histological asbestosis can occur at lower exposures [22]. As discussed by Henderson et al. [17,101], others have claimed that the dose necessary for the development of asbestosis is 25–100 fiber-yrs [101]. The estimated cumulative dose of asbestos required for induction of asbestosis has diminished over the years, and reference [102] refers to a lifetime risk of asbestosis of 2/1000 at 4.5 fiber-yrs, drawing attention to ‘a few’ asbestosis deaths at less than five fiber-yrs in the study reported by Dement et al. [103]. In their stepwise decision-tree approach to assessing asbestosis, Burdorf and Swuste [102] suggested that for any probability of exposure defined by industry, evidence of direct exposure at a level of ≥5.0 fibers/mL for more than one year is sufficient for ‘ascertainment’ of asbestosis (i.e., >5.0 fiber-yrs). When assessing asbestosis related to low-’dose’ exposures, it is important for two factors to be taken into account: (i) a low mean/median ‘dose’, as calculated across a population from average airborne fiber concentrations, may not reflect large variations in exposure for some individuals comprising the relevant population; and (ii) the nonrecognition of other exposures [17].

A cumulative asbestos exposure of 25 fiber-yrs is also the level accepted as being associated with an approximate doubling of lung cancer risk relative to a nonexposed person i.e., additive increase in rate (risk) ratio (RR) from 1 to 2 for mixed fiber type exposures according to De Vuyst [104]. This was the basis of its selection by the German authorities [105] and the original Helsinki expert group [46]. Other studies would put the additive increase in rate ratio for 25 fiber-yrs at 0.06, with meta-analysis of all fiber types included [106], 0.25, UK chrysotile textile work [101], or 0.5, USA chrysotile textile work [107,108].

A rate ratio of RR = 2 has been commonly equated by some with an attributable fraction in the exposed (AF_E_), corresponding to ‘probability of causation’) of 0.5 (50%). Thus, if RR = (incidence rate exposed) ÷ (incidence rate unexposed) = I_E_/I_0_ = 2, then AF_E_ is given by (I_E_ − I_0_)/I_E_ = (RR − 1)/RR = (2 − 1)/2 = 0.5. The ratio (I_E_ − I_0_)/I_E_ represents the proportion of diseased cases in the exposed group in which disease is caused by exposure (some cases would appear in the absence of exposure). If AF_E_ = 0.5, the proportion of exposed cases in whom the exposure caused the disease would be 0.5 (50%).

Translated into a probability in the individual, one could thus argue that given an exposed case, the probability that the exposure caused the disease is 0.5 (50%). This could be conveniently equated with the civil standard of proof. However, there has been persistent, strong criticism of this approach. It is thought that the attributable fraction in the exposed may often underestimate the probability of causation. This is because the probability of causation applies to an individual, and depends on biological, epidemiological, and other evidence, whereas AF_E_ is an epidemiological measure and applies to populations. Sampling error, variation in background risk, individual susceptibility, and biological mechanisms can all affect the probability of causation. Furthermore, there is a distinction between cases solely caused by exposure and those whose causation is accelerated by exposure; for expositions of these concepts in mathematical, semilay and legally-directed terms, see [72,109,110,111,112]

Greenland, for example, pointed out two misconceptions, which are common in the literature relating epidemiology to compensation decisions. First, “the probability of causation cannot be computed solely from the relative risk.” Second, “the exposure dose at which the probability of causation exceeds 50% (the point at which exposure causation is more likely than not) may fall well below the “doubling dose” (the dose at which the incidence of disease is doubled).” Greenland concluded: “In particular, and contrary to common perceptions, a rate fraction of 50% (or equivalently, a rate ratio of 2) does not correspond to a 50% probability of causation.” [110].

In the case of some occupational lung cancers, a RR = 1.1 (rather than 2), equating to an attributable fraction of 9%, has been accepted as indication of a material liability to causation rather than ‘the cause of’ by civil courts [113,114].

The IARC classifies carcinogens as category 1 (human carcinogen) when the typical rate ratios from human cohort studies are 1.3–1.6, for example silica [115], and diesel exhaust fumes [116]. The caveats on the well-known Bradford Hill guidelines [117] on causality are laid out in Rothman and Greenland [118], where the relation of magnitude of association (e.g., magnitude of rate ratio) to reliability of causal inference is discussed, giving examples of low rate ratios where causation is accepted (e.g., smoking and cardiovascular disease; passive smoking and lung cancer). There is nothing particularly magical about a RR = 2 as compared to, say, RR = 1.1. A value of RR = 2 represents a doubling, whereas a RR = 1.1 represents an increase in rate ratio by 10% [110].

Whether a RR = 1.1 is clinically-significant or not depends on the size of the exposed group and the background (nonexposed) incidence rate. For example, if 100,000 persons are exposed to an agent at an exposure level creating a rate ratio of 3, when the background incidence rate is 1 per 100,000 per year, then 3 − 1 = 2 extra cases per year are produced in this population by the exposure. If 100,000 persons are exposed to an agent at an exposure level creating a rate ratio of 1.1 when the background incidence rate is 70/100,000 per year (as for lung cancer), then 77 − 70 = 7 extra cases per year are produced in this population. It is important to be sure that a rate ratio of 1.1 obtained from an epidemiological study is statistically significant (i.e., not due to sampling error or chance), and that it is not the result of uncontrolled confounding factors. This is ensured by good epidemiological study design. It is now generally thought that those wishing to challenge increased rate ratios of low magnitude on the basis of unknown confounders should bear the onus of demonstrating this confounding effect. It remains generally true, however, that higher rate ratios are less likely to be distorted by unknown confounders.

The clinical or public health significance of an increased rate ratio also depends on the proportion of the population exposed. If many in a population are exposed at a low RR, this can result in a larger number of exposure-attributable cases in the total population compared to a situation where there is a high RR but only a small number of exposed.

The lung cancer RR for 25 fiber-yrs of exposure has been estimated at 2.5 for amosite factory workers [119] and 1.8 for the Wittenoom crocidolite miners/millers in Western Australia [17]. Rödelsperger and Woitowitz also found that an analysis of South African amosite and crocidolite miner data indicated rate ratios of 2 at less than 25 fiber-yrs of cumulative exposure [120].

From the year 2000, three important new studies relating to the risk-dose relationship for asbestos-related lung cancer have been published. These studies are of mixed exposures, and are well designed case referent studies. In a very large, very well designed case-referent study in Sweden (1042 cases 2364 referents), the coefficient for increase in lung cancer risk was 0.14 per fiber-yr, giving a RR = 4.5 for 25 fiber-yrs [28]. In a further study [29] exploring risk at lower ‘doses’, the lung cancer risk was 1.9 at a ‘dose’ of 4 fiber-yrs. A natural spline model applied to 19 studies estimated RR for exposure of 4 and 40 fiber years between 1.013 1and 1.027 and 1.13 and 1.3, respectively, but was heavily influenced by three-to-four-fold differences in RR between chysotile and amphibole at doses below 40 fiber years [121]. The effect of smoking was between additive and multiplicative. In June 2002, in a large population-based matched, case-referent study (839 cases 839 referents), Pohlabeln et al. [122] found in a validation subsample of 164 male cases and their 164 referents a smoking adjusted odds ratio (OR) of 1.71 (95% CI = 1.18–2.46) for an asbestos exposure of 25 fiber-yrs and a smoking-adjusted risk (OR) of 1.94 (95% CI = 1.10–3.43) for the category >10 fiber-yrs. The authors claimed that these results were consistent with a doubling of lung cancer risk at 25 fiber-yrs of exposure.

The Olsson [43] pooled case referent study would give a doubling of risk at of 16.4 fiber-yrs (all men) and 30.3 fiber-yrs (blue collar workers)

Case-referent studies, if well designed, give the same information as cohort studies, and should be included in any meta-analyses along with all relevant studies, published or unpublished [118].

A meta-analysis of 17 selected published cohort studies by Hodgson and Darnton [31] set forth a lung cancer rate ratio of 0.05 per fiber-yr for amphibole asbestos (RR = 2.25 at 25 fiber-yrs) and 0.001 per fiber-yr for commercial chrysotile (RR = 1.025 at 25 fiber-yrs) as reasonable overall best estimates. The highest estimate for the chrysotile rate ratio was 0.005 (RR = 1.125 at 25 fiber-yrs). Mixed exposures would have intermediate values. However, Hodgson and Darnton [31] omitted the chrysotile cohort study with the highest risk/dose coefficient (South Carolina textile workers) from their analysis, and they also excluded case-referent studies. Inclusion of those studies would have resulted in higher overall chrysotile textile and mixed exposure coefficients. More recent analyses of relative lung cancer potency of different fiber types using a similar selection of cohorts (but with some significant exclusions) by Berman and Crump [123] give similar results. The US EPA has now decided not to use the Berman and Crump modeling in risk assessment. Recent reevaluations of the relative lung cancer carcinogenicity of chrysotile and the amphiboles, taking data quality into account, concluded that there is little difference between chrysotile and amphibole in this respect [124]. A recent nested case-referent study within a major Chinese cohort study of workers exposed only to chrysotile gave an OR = 3.7 in the high exposure group, and more than additive synergism with smoking [125].

There is no threshold for asbestos related lung cancer [55,126]. Some studies appear to indicate that adenocarcinoma risk tends to have a steeper dose-response gradient with quantified asbestos exposure than other lung cancer cell types [126]. A recent, very large general population cohort study (58,279 men followed for 17.3 years) of cancer risks, especially at lower levels of occupational asbestos exposure, appeared to show that lung adenocarcinoma is only a significant risk at the highest level of exposure studied [127]. (This finding contrasts with the preceding statement that adenocarcinoma has a steeper dose response relationship with asbestos.) However, in their discussion (p. 17), the authors explain that this may be due to the study design and the fact that smoking is only weakly associated with adenocarcinoma. When stratified for smoking, the asbestos association for adenocarcinoma is indeed higher than for all cell types pooled. It should also be noted that the highest cumulative exposure category in this study is only 6.7 fiber-yrs (median).

The deficiencies and uncertainties of the meta-analyses of the published data with large heterogeneity have been pointed out [128].

Further discussion of more recent literature on asbestos and lung cancer in relation to the Helsinki and AWARD Criteria has been provided [1,30,129]. Those reviews defend the epidemiological bases of the Helsinki Criteria and strengthen them in the light of new data since 1997. They defend the position that asbestosis is not a prerequisite for attribution, and that for mixed fiber type exposure, where the precise composition is not known, the 25 fiber-yr level (or equivalent exposure history or lung fiber content) is a reasonable level for attributing lung cancer to asbestos exposure, irrespective of smoking history. They accept that for certain cohorts, for lifelong nonsmokers or where the actual type of exposure is known with certainty, then a higher or lower criterion may apply. For lifelong nonsmokers or smokers who have quit for more than 30 years before diagnosis, a cumulative exposure of 5 fiber-yrs has been recommended as sufficient to assign a causal liability by asbestos to lung cancer [30,37]. Lifelong nonsmokers have about three times the dose-related risk of lung cancer from asbestos as smokers [130]. Guidotti [89] considers that because of the rarity of lung cancer in nonsmokers, an association of lung cancer with asbestos can be assumed if any asbestos exposure has occurred. This could be in addition to a coaction from other occupational lung carcinogen exposure [131]. Asbestosis—not asbestos exposure—has been invoked as the ‘primary risk factor for lung cancer’ as recently as 2014 [38], and Cagle [51] commented that ‘Unless and until a better marker comes along, the only consistently reliable marker for an asbestos-related lung cancer is asbestosis, especially in asbestos workers who are also tobacco smokers.’ We consider that in some instances, a clinical-radiologic diagnosis of early/mild asbestosis cannot be made with consistency or reliability, and may be the subject of dispute among respiratory physicians and radiologists, because of the following disagreements:

- Dispute as to whether there is genuine interstitial fibrosis, even in high-resolution CT scans [132], or whether any changes are related to associated pleural fibrosis.
- Dispute as to whether interstitial fibrosis represents asbestosis or idiopathic pulmonary fibrosis (usual interstitial pneumonia [UIP]) or nonspecific interstitial pneumonia [NSIP] unrelated to asbestos, especially when pleural plaques are not demonstrable [133].
- Even on histologic examination, disagreement between pathologists as to whether there is genuine interstitial fibrosis in a distribution appropriate for asbestosis, i.e., a UIP pattern or the fibrotic variant of NSIP, or diffuse interstitial fibrosis which is not readily classifiable as either [133], or whether there are sufficient asbestos bodies for that diagnosis [134].

We consider that the prevailing evidence conforms to the cumulative exposure model for the risk of lung cancer with no requirement for asbestosis (although asbestosis remains one criterion for asbestos exposure). In addition, a recent systematic literature analysis of 5864 citations on asbestos and lung cancer in PubMed and Embase generally confirms the principles of the Helsinki Criteria [36].

### 3.6. Problems with Numerical Assessments of Asbestos Exposure

Systematic measurements of airborne asbestos fiber concentrations in various workplaces have been carried out in nations such as Germany [105] and Sweden [28,29], and in special industries elsewhere, thereby allowing estimates to be made of cumulative exposure expressed as fibers/mL-years. However, no such measurements have been carried out in many workplaces in various nations —especially for the end-uses of asbestos-containing materials (e.g., building construction; shipyards, and power stations in Australia). In these latter circumstances, estimates of exposure are often assessed by the use of data from other workplaces and nations. In adversarial court proceedings, we have often encountered widely-divergent estimates of exposure (sometimes more than 100-fold) on the same case, from occupational hygienists, some incompatible with the diseases present (e.g., in one case of a pleural mesothelioma patient with asbestosis and a 10-year history of ship construction in Scotland and Australia, his estimated cumulative exposure was about 1.0 fiber-yr). In such circumstances, we prefer to use expressions such as ‘light’, ‘moderate’, or ‘heavy’ exposure, while acknowledging the imprecision of those terms; at least they are not predicated upon the specious pseudo-precision of a numerical estimate.

The background levels of asbestos bodies in the lungs of patients with no specific asbestos exposure seem to be higher in Finland than in other countries. Karjalainen et al. [44] analyzed the relation between pulmonary concentrations of asbestos bodies (AB) and asbestos fibers (AF) from lung cancer patients in Helsinki as indicators of asbestos exposure. The regression equation log (AF) = −0.429 + 0.600 log (AB) was found to predict the concentration of asbestos fibers (10^6^ fibers · g^−1^) corresponding to a given number of asbestos bodies in a section of lung tissue. In medico-legal cases, the methodological variation involved in asbestos fiber and asbestos body counting must be recognized, and all available exposure data should be used to produce the best possible estimate of exposure.

In terms of the lung tissue concentration of uncoated asbestos fiber concentrations for mixed-fiber exposures, the Helsinki Criteria set forth counts of ≥2.0 million amphibole fibers (>5 μm in length)/g dry lung or ≥5.0 million amphibole fibers >1 μm in length. However, because of different methodologies, different laboratories can find different fiber concentrations [36]. Therefore, we would now modify this criterion and specify an amphibole fiber count above the fifth percentile concentration for cases of asbestosis, as assessed by the same laboratory.

### 3.7. Proposed Criteria for Attribution of Lung Cancers to Exposure to Asbestos

A set of criteria for attribution with respect to asbestos from the original Helsinki Criteria as been set forth by Henderson and Leigh [30], which can be presented in slightly modified and abbreviated form for the smoking categories as follows

Asbestos exposure with a minimum latency interval of 10 years

AND

For current smokers:

A nondisputed or majority clinical-radiologic or histologic diagnosis of asbestosis.

OR

The occurrence of asbestosis among other workers in the same workforce carrying out similar work for similar durations of time and at similar times.

OR

A nondisputed/majority estimate of cumulative exposure to asbestos of 25 fiber-years or more for mixed-fiber, end-use exposure to asbestos (e.g., in the building construction industry and for insulation work). For amphibole-only (amosite or crocidolite) exposures, a nondisputed estimated cumulative exposure of 20 or 25 fiber-years, and 25 fiber-years for asbestos textile workers. For chrysotile-only exposures, most notably the Canadian chrysotile miners and millers, and exposure to friction products, 200 fiber-years, and for other chrysotile-only exposure, 100 fiber years. This is based on the estimated relative potency of 1:4 amphibole:chrysotile [121].

OR

At least 5 years of asbestos exposure before 1975, or 5–10 years after 1975, for asbestos textile workers, asbestos insulation workers including work in shipbuilding, power stations, railways workshops, and others in close proximity to such work, especially when it was carried out in confined and poorly ventilated workplaces, or a duration of one year for work that involved consistent or frequent spraying of asbestos insulation. Henderson and Leigh [30] excluded Canadian chrysotile miners/millers and friction products workers from this assessment.

OR

For never-smokers or those who had ceased smoking 30 years or more before the diagnosis of lung cancer; cumulative exposure amounting to 5 fiber-years, or exposure amounting to one-third of the durations for work set forth in the preceding paragraph.

OR

A concentration of asbestos bodies or uncoated amphibole fibers at or in excess of the fifth percentile count in cases of asbestosis for the same laboratory (for fibers of the same length) for mixed-fiber end-use exposures. Because chrysotile fibers are cleared from lung more rapidly than amphibole, fiber assays should not be used for chrysotile-only exposures; instead, the occupational history should be substituted.

These criteria are summarized in a simplified flow chart in Figure 2.

The proposed Criteria are designed primarily for statutory compensation where smoking is not to be considered. In a litigation situation where tobacco smoking can be considered, either as joint tortfeasor or as an example of contributory negligence, different approaches may be needed, and apportionment methods applied. A simplified flowchart is presented in Figure 2. Since asbestos and smoking are inseparable agents at the biological level in the individual case, there will always be some joint contribution to causation by the asbestos, even at exposures below 25 fiber/mL-yr; this should be proportionally compensated, taking smoking history and any possible genetic susceptibility into account. This has also been recognized in a recent critique of the 2014 update of the Helsinki criteria [135]. A range of possible models for taking smoking into account is available [12,89].

However, it should be emphasized that all such apportionment schemes are contrived mathematical models, even if based on both biometric theory and empirical data, and cannot reflect perfectly the complex biological reality. The possibility of taking genetic susceptibility into account in individual lung cancer cases may become more feasible with the application of genome-wide gene-environment interaction analyses for asbestos exposure [136].

## 4. Conclusions

The new knowledge gained through this predominantly didactic review takes the form of evidence for the synergism between asbestos and tobacco smoke in lung cancer causation at a biological level, and the proposed science-based and equitable approach to compensation implications for asbestos-related lung cancer. The evaluated statistical data conform to a multiplicative model for lung cancer risk, with no requirement for the presence of asbestosis.

## Appendix A

### Definitions of Additive and Multiplicative Models

*Additive model (1)* A model used in epidemiology, the structural form of which implies that the disease rate (R) is a linear function of an intercept term (α), an exposure effect (βE), and covariate effects (δC): R = α + βE + δC. Expressed equivalently, the rate difference (RD) for the effect of a unit increase in exposure is constant across covariate levels and equal to the exposure coefficient β: RD = β. For example, the joint effect of two dichotomous exposures E_1_ and E_2_ (coded 1 = exposure present, 0 = exposure absent) on the rate difference is the sum of their singular effects:

RD = [α + β_1_(E_1_ = 1) + β_2_(E_2_ = 1) + δC] ‒ [α + β_1_(E_1_ = 0) + β_2_(E_2_ = 0) + δC] = β_1_ + β_2_ = RD_1_ + RD_2_ (A1)

Hence the term additive model.

*Multiplicative model (2)* A model used in epidemiology, the structural form of which implies that the disease rate (R) is an exponential function of an intercept term (α), an exposure effect (βE), and covariate effects (δC): R = exp(α + βE + δC). Expressed equivalently, the rate ratio (RR) for the effect of a unit increase in exposure is constant across covariate levels and equal to the antilog of the exposure coefficient β: RR = exp(β). For example, the joint effect of two dichotomous exposures E_1_ and E_2_ (coded 1 = exposure present, 0 = exposure absent) on the rate ratio is the product of their singular effects:

RR = exp[α + β_1_(E_1_ = 1) + β_2_(E_2_ = 1) + δC]/exp[α + β_1_(E_1_ = 0) + β_2_(E_2_ = 0) + δC] = exp(β_1_ + β_2_) = exp(β_1_) × exp(β_2_) = RR_1_ × RR_2_ (A2)

Hence the term multiplicative model.
